# Antimicrobial use in adult patients receiving extracorporeal membrane oxygenation in a center without routine prophylaxis

**DOI:** 10.1017/ash.2026.10834

**Published:** 2026-09-09

**Authors:** James Riesing, Erika O’Neil, James Aden, Kayla Scheps, Joseph Marcus

**Affiliations:** 1 Brooke Army Medical Center, USA; 2 Department of Pediatrics, Uniformed Services University of the Health Science, USA; 3 Infectious Diseases, Department of Medicine, Uniformed Services University of the Health Science, San Antonio, USA

## Abstract

Antimicrobial use in patients receiving extracorporeal membrane oxygenation (ECMO) is poorly described. In this study, evaluating antimicrobial use at a single center without ECMO-specific antimicrobial prophylaxis protocols, antimicrobials were prescribed on 85% of ECMO days. Targeted antimicrobial days (52%) were more common than empiric (29%) or non-ECMO prophylaxis (4%) days.

## Introduction

One of the most common complications in patients receiving extracorporeal membrane oxygenation (ECMO) is the development of healthcare-associated infections, which are associated with significant morbidity and mortality.^1^ To prevent infection, a variety of strategies are used, including prophylactic antibiotics, which are used in roughly half of ECMO centers.^2^ While the use of narrow-spectrum prophylactic antibiotics has been associated with the decreased use of broad-spectrum antibiotics, data is mixed on their utility in decreasing infections or improving patient outcomes.^3,4^

With the global efforts for antimicrobial stewardship on ECMO,^5^ there is a need to better characterize antimicrobial use in patients receiving ECMO in all settings. In this study, we describe antimicrobial use at a single center without any ECMO-specific protocols for antimicrobial prophylaxis.

## Methods

This retrospective cross-sectional analysis evaluated systemic antimicrobial use in adult patients receiving ECMO for greater than 24 hours at Brooke Army Medical Center (BAMC) between January 1, 2022 and February 29, 2024. In addition to serving as the sole ECMO center in the United States Military Health System, BAMC also cares for civilian patients across Texas as an ECMO referral center and as a Level 1 trauma center. There are no solid organ transplant capabilities at this facility; patients who require a bridge to transplant are ultimately transferred to other facilities. At this facility, both venovenous (VV) and venoarterial (VA) ECMO is used. There are currently no standardized systemic antimicrobial protocols at this center for patients receiving ECMO, and all antibiotics are given due to clinical need or for prophylaxis for non-ECMO indications, such as neutropenia or surgical prophylaxis.

In this study, antimicrobial use was defined as any systemic antimicrobial received on a given calendar day while a patient was on the ECMO circuit. Targeted antimicrobials were defined as those used in patients with a suspected infection and a positive culture. Empiric antimicrobials were defined as antimicrobials used in patients with a suspected infection without positive cultures. Prophylaxis was defined as antimicrobials that were used to prevent future infections associated with immunosuppressive medications, perioperative prophylaxis or severe neutropenia. If a patient had multiple indications, such as targeted, empiric, or prophylaxis, targeted was selected as the primary indication that day. If a patient had an empiric indication, but also prophylaxis, the day was classified as an empiric day.

Descriptive statistics were assumed to be non-parametric and described with medians and interquartile ranges (IQR). All analysis was performed using JMP v13.2 (SAS Corp, Cary NC). This protocol was reviewed by the San Antonio Institutional Review Board (protocol number: 24-20942) and determined to be exempt and consent was not required from subjects.

## Results

During the study period, sixty-five patients received a total of 831 days of ECMO (median: 10 d [IQR 4–21]). The most common configuration in these patients was VV (81.5%), with the remaining patients receiving VA ECMO or a hybrid modality. Indications for ECMO were generally due to medical chief complaints (58.5%), followed by trauma (21.5%), cardiac (13.8%), and burn injuries (6.2%). Forty-nine (75.4%) patients survived hospital discharge.

**Figure 1.**
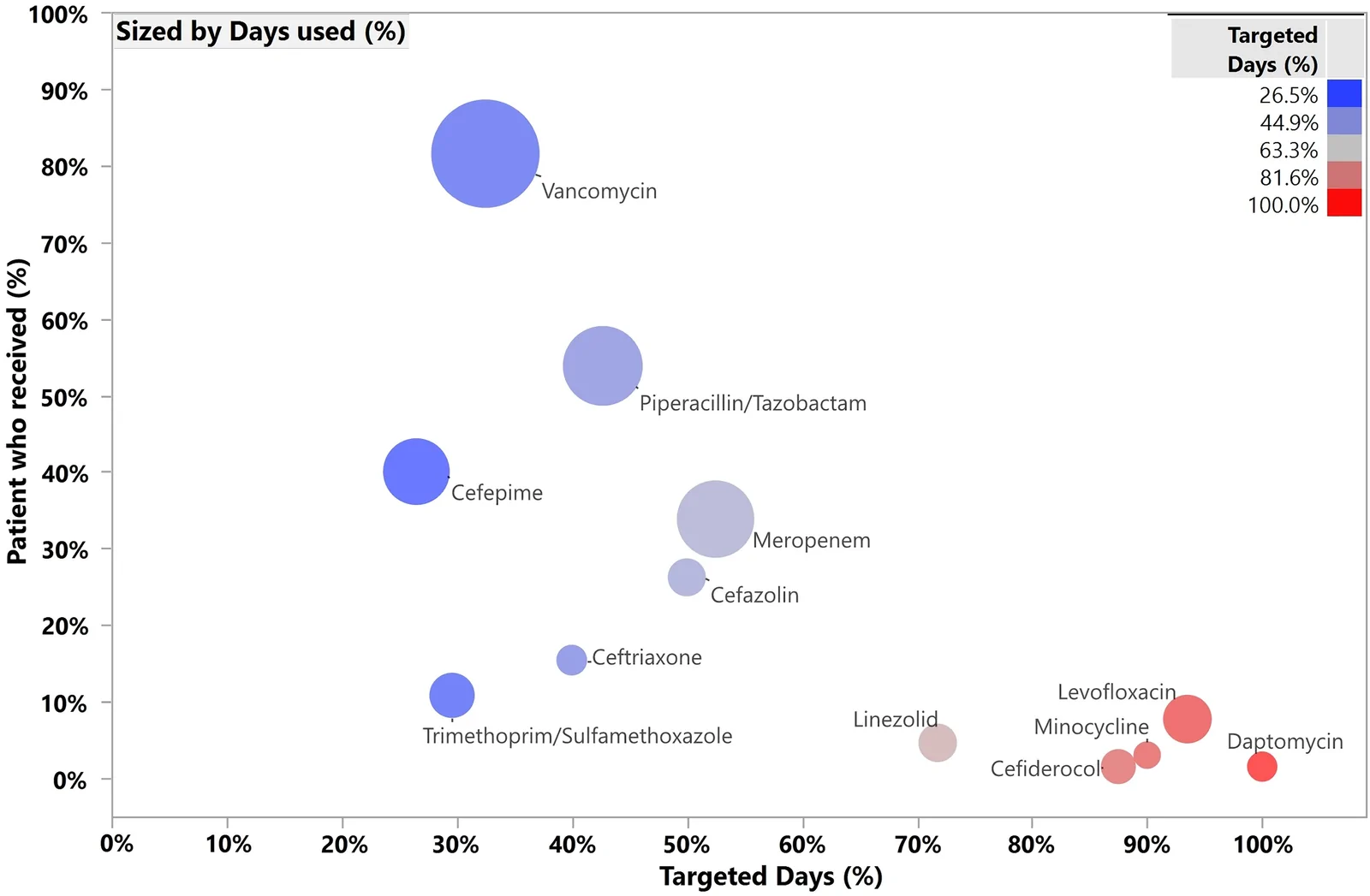
Bubble plot of antibiotic use in adult patients receiving ECMO at Brooke Army Medical Center between January 1, 2022 and February 29, 2024. The size of bubble correlates with days of therapy.

Of the patients who received ECMO during the study period, 64 (98.5%) received an antimicrobial on 706 (85.0%) ECMO days (Figure 1, Table 1). The majority (52.0%) of antimicrobial days were targeting a specific pathogen that grew on culture, while 28.8% of antimicrobial days had empiric antibiotics prescribed, and 4.2% of days were for a non-ECMO indication for prophylaxis. Of 239 empiric therapy days, 136 (56.9%) were within 72 hours of a clinical change and 103 (43.1%) were antibiotic days continued beyond 72 hours after a clinical change. The most common syndrome treated was respiratory infections with 29 targeted treatments during the study period with methicillin-susceptible *Staphylococcus aureus* (n = 4, 14.0%) and *Klebsiella pneumoniae* (n = 3, 10.0%) being the two most common organisms. The most common fungal respiratory infection was *Aspergillus* (n = 2, 6.9%) The second most common syndrome treated was blood stream infections (n = 26) with *Staphylococcus epidermidis* (n = 8, 30.7%), methicillin-susceptible *S. aureus* (n = 4, 15%), and *Enterococcus faecalis* (n = 3, 11.5%) being the most common isolated organisms. There were few skin and soft tissue infections (n = 5) and urinary tract infections (n = 2) treated in this cohort.

**Table 1.** Antimicrobial use at Brooke Army Medical Center between January 1, 2022 and February 29, 2024 Table 1 long description.

Antimicrobial	Days used (%)	Patient who received (%)	Targeted days (%)	Empiric days (%)	Prophylactic days (%)
Vancomycin	308 (37.1)	53 (81.5)	100 (32.5)	202 (65.5)	6 (2)
Piperacillin/Tazobactam	168 (20.2)	35 (53.8)	72 (42.7)	96 (57.1)	2 (1.2)
Meropenem	158 (19.0)	22 (33.8)	83 (52.5)	75 (47.6)	0 (0)
Cefepime	117 (14.1)	26 (40)	31 (26.5)	84 (71.8)	2 (1.7)
Micafungin	80 (9.6)	12 (18.5)	60 (75)	20 (25)	0 (0)
Amphotericin	78 (9.4)	3 (4.6)	77 (98.7)	1 (1.3)	0 (0)
Levofloxacin	62 (7.5)	5 (7.7)	58 (93.5)	4 (6.5)	0 (0)
Trimethoprim/Sulfamethoxazole	54 (6.5)	7 (10.8)	16 (29.6)	23 (42.6)	15 (2.8)
Linezolid	39 (4.7)	3 (4.6)	28 (71.8)	11 (28.2)	0 (0)
Cefazolin	38 (4.6)	17 (26.2)	19 (50)	2 (5.3)	17 (44.7)
Fluconazole	34 (4.1)	3 (4.6)	24 (70.6)	5 (14.7)	5 (14.7)
Cefiderocol	32 (3.9)	1 (1.5)	28 (87.5)	3 (9.4)	1 (3.1)
Isavuconazole	28 (3.4)	5 (7.7)	26 (92.9)	2 (71.4)	0 (0)
Ceftriaxone	25 (3.0)	10 (15.4)	10 (40)	9 (36)	6 (24)
Daptomycin	24 (2.9)	1 (1.5)	24 (100)	0 (0)	0 (0)
Minocycline	20 (2.4)	2 (3.0)	18 (90)	2 (10)	0 (0)
Voriconazole	19 (2.3)	4 (6.2)	19 (100)	0 (0)	0 (0)
Ganciclovir	1 (0.1)	1 (1.5)	1 (100)	0 (0)	0 (0)
Total			432 (52)	239 (28.8)	35 (4.2)

The most common antimicrobial prescribed was vancomycin for 308 (37.0%) days in 53 (81.5%) patients, piperacillin/tazobactam for 168 (20.2%) days in 35 (53.8%) patients, and meropenem for 158 days (19.0%) in 22 (33.8%) patients. The most used antifungal was micafungin for 80 days (9.6%) in 12 (18.5%) patients. Most vancomycin (65.5%) and piperacillin/tazobactam (57.1%) use was empiric. The majority of meropenem (52.5%) and micafungin (75.0%) use was targeted. Amongst the 35 antimicrobial days for prophylaxis, the most common agents prescribed were cefazolin (17 d) and trimethoprim-sulfamethoxazole (15 d).

## Discussion

In this single-center retrospective study, we evaluated systemic antimicrobial use in patients cannulated for ECMO between 2022 and 2024. Despite no ECMO-specific antimicrobial prophylaxis protocol, antimicrobials were used on 85% of ECMO days. While most days of antimicrobials were written in response to a positive culture, nearly a third were empiric therapy initiated due to clinical changes. These findings suggest that antimicrobial exposure during ECMO is substantial and the frequent continuation of empiric therapy beyond 72 hours is an opportunity for future stewardship interventions.

There is limited reported data on the frequency of antimicrobial use in patients receiving ECMO, but it has been demonstrated that antimicrobial use is frequent in other critically ill patients. For example, an Ontario population-based cohort noted that there were 624 days of therapy per 1,000 intensive care days with 56% of patients receiving antibiotics with the highest rates in medical/surgical intensive care units (697 d of therapy per 1,000).^6^ Our study demonstrated a significantly greater amount of antimicrobial use. This could be due to either the greater disease severity in patients receiving ECMO or a higher burden of infections.

With the varied approaches to antimicrobial prophylaxis performed at ECMO centers, the specific agents used are rarely described. The most reported prophylactic agents are first-generation cephalosporins, due to its favorable pharmacokinetic parameters.^3,7,8^ Narrow-spectrum antibiotics, such as cefazolin, were rarely prescribed in this cohort and more than half of the empiric antimicrobial days included a broad-spectrum agent. This prolonged empiric use of broad-spectrum should be a target of stewardship initiatives with either forced de-escalation protocols or ECMO infectious disease rounds.

There are several limitations to the data. First, this was a retrospective study with multiple patients transferred from outside centers. It is possible that some of the patients were misclassified as receiving empiric antibiotics when they were targeting an organism from an outside facility. Additionally, we only evaluated ECMO usage at a single center with a high proportion of trauma and burn patients compared to other ECMO centers. Finally, positive cultures were treated as true infections. This is particularly concerning as *Staphylococcus epidermidis* was the most common cause of bacteremia, even though it is frequently a colonizing organism in patients receiving ECMO.^9^

Antimicrobial usage in patients receiving ECMO is poorly reported. In this single-center, retrospective study, we found that, even without a prophylaxis protocol, patients received antimicrobials on most days. Empiric therapy was frequent, often prolonged, and presents an opportunity for antimicrobial stewardship interventions. Further evaluation with multicenter, outcome-driven studies are needed to optimize antimicrobial use in ECMO.

## Research transparency

Data is available on request to the corresponding author.

## Table 1. Long description

The table presents data on antimicrobial use among patients who received ECMO between January 1, 2022, and February 29, 2024. It includes columns for the type of antimicrobial, days used, percentage of patients who received the antimicrobial, targeted days, empiric days, and prophylactic days. The table has 20 rows and 6 columns. Row 1: Vancomycin, 308 days (37.1 percent), 53 patients (81.5 percent), 100 targeted days (32.5 percent), 202 empiric days (65.5 percent), 6 prophylactic days (2 percent). Row 2: Piperacillin/Tazobactam, 168 days (20.2 percent), 35 patients (53.8 percent), 72 targeted days (42.7 percent), 96 empiric days (57.1 percent), 2 prophylactic days (1.2 percent). Row 3: Meropenem, 158 days (19.0 percent), 22 patients (33.8 percent), 83 targeted days (52.5 percent), 75 empiric days (47.6 percent), 0 prophylactic days (0 percent). Row 4: Cefepime, 117 days (14.1 percent), 26 patients (40 percent), 31 targeted days (26.5 percent), 84 empiric days (71.8 percent), 2 prophylactic days (1.7 percent). Row 5: Micafungin, 80 days (9.6 percent), 12 patients (18.5 percent), 60 targeted days (75 percent), 20 empiric days (25 percent), 0 prophylactic days (0 percent). Row 6: Amphotericin, 78 days (9.4 percent), 3 patients (4.6 percent), 77 targeted days (98.7 percent), 1 empiric day (1.3 percent), 0 prophylactic days (0 percent). Row 7: Levofloxacin, 62 days (7.5 percent), 5 patients (7.7 percent), 58 targeted days (93.5 percent), 4 empiric days (6.5 percent), 0 prophylactic days (0 percent). Row 8: Trimethoprim/Sulfamethoxazole, 54 days (6.5 percent), 7 patients (10.8 percent), 16 targeted days (29.6 percent), 23 empiric days (42.6 percent), 15 prophylactic days (2.8 percent). Row 9: Linezolid, 39 days (4.7 percent), 3 patients (4.6 percent), 28 targeted days (71.8 percent), 11 empiric days (28.2 percent), 0 prophylactic days (0 percent). Row 10: Cefazolin, 38 days (4.6 percent), 17 patients (26.2 percent), 19 targeted days (50 percent), 2 empiric days (5.3 percent), 17 prophylactic days (44.7 percent). Row 11: Fluconazole, 34 days (4.1 percent), 3 patients (4.6 percent), 24 targeted days (70.6 percent), 5 empiric days (14.7 percent), 5 prophylactic days (14.7 percent). Row 12: Cefiderocol, 32 days (3.9 percent), 1 patient (1.5 percent), 28 targeted days (87.5 percent), 3 empiric days (9.4 percent), 1 prophylactic day (3.1 percent). Row 13: Isavuconazole, 28 days (3.4 percent), 5 patients (7.7 percent), 26 targeted days (92.9 percent), 2 empiric days (7.1 percent), 0 prophylactic days (0 percent). Row 14: Ceftriaxone, 25 days (3.0 percent), 10 patients (15.4 percent), 10 targeted days (40 percent), 9 empiric days (36 percent), 6 prophylactic days (24 percent). Row 15: Daptomycin, 24 days (2.9 percent), 1 patient (1.5 percent), 24 targeted days (100 percent), 0 empiric days (0 percent), 0 prophylactic days (0 percent). Row 16: Minocycline, 20 days (2.4 percent), 2 patients (3.0 percent), 18 targeted days (90 percent), 2 empiric days (10 percent), 0 prophylactic days (0 percent). Row 17: Voriconazole, 19 days (2.3 percent), 4 patients (6.2 percent), 19 targeted days (100 percent), 0 empiric days (0 percent), 0 prophylactic days (0 percent). Row 18: Ganciclovir, 1 day (0.1 percent), 1 patient (1.5 percent), 1 targeted day (100 percent), 0 empiric days (0 percent), 0 prophylactic days (0 percent). Row 19: Total, 432 targeted days (52 percent), 239 empiric days (28.8 percent), 35 prophylactic days (4.2 percent).

Navigate back to Table 1..
